# Research, evidence and policymaking: the perspectives of policy actors on improving uptake of evidence in health policy development and implementation in Uganda

**DOI:** 10.1186/1471-2458-12-109

**Published:** 2012-02-09

**Authors:** Juliet Nabyonga Orem, David Kaawa Mafigiri, Bruno Marchal, Freddie Ssengooba, Jean Macq, Bart Criel

**Affiliations:** 1 WHO Uganda office, Health systems and services cluster, P. O. Box 24578, Kampala, Uganda; 2 Makerere University School of Social Sciences, P.O Box 7072, Kampala, Uganda; 3 Institute of Tropical Medicine Antwerp-Belgium, Nationalestraat 155, 2000 Antwerp, Belgium; 4 School of Public Health, Makerere University, P.O. Box. 7072, Kampala, Uganda; 5 Université Catholique de Louvain, Boite 3058; Clos Chapelle aux champs, 30, 1200 Bruxelles, Belgium

**Keywords:** Research, Policy, Practice, Implementation gap, Uganda, Low income countries

## Abstract

**Background:**

Use of evidence in health policymaking plays an important role, especially in resource-constrained settings where informed decisions on resource allocation are paramount. Several knowledge translation (KT) models have been developed, but few have been applied to health policymaking in low income countries. If KT models are expected to explain evidence uptake and implementation, or lack of it, they must be contextualized and take into account the specificity of low income countries for example, the strong influence of donors. The main objective of this research is to elaborate a Middle Range Theory (MRT) of KT in Uganda that can also serve as a reference for other low- and middle income countries.

**Methods:**

This two-step study employed qualitative approaches to examine the principal barriers and facilitating factors to KT. Step 1 involved a literature review and identification of common themes. The results informed the development of the initial MRT, which details the facilitating factors and barriers to KT at the different stages of research and policy development. In Step 2, these were further refined through key informant interviews with policymakers and researchers in Uganda. Deductive content and thematic analysis was carried out to assess the degree of convergence with the elements of the initial MRT and to identify other emerging issues.

**Results:**

Review of the literature revealed that the most common emerging facilitating factors could be grouped under institutional strengthening for KT, research characteristics, dissemination, partnerships and political context. The analysis of interviews, however, showed that policymakers and researchers ranked institutional strengthening for KT, research characteristics and partnerships as the most important. New factors emphasized by respondents were the use of mainstreamed structures within MoH to coordinate and disseminate research, the separation of roles between researchers and policymakers, and the role of the community and civil society in KT.

**Conclusions:**

This study refined an initial MRT on KT in policymaking in the health sector in Uganda that was based on a literature review. It provides a framework that can be used in empirical research of the process of KT on specific policy issues.

## Background

Use of evidence in health policymaking and health systems development plays an important role in guiding investment decisions, improving service delivery and health outcomes, even more so in resource constrained settings where informed investment decisions are paramount [1,2]. Over the last decade, much attention has been paid to this process. Several terminologies have been used for uptake of evidence including Knowledge Translation (KT), research utilization, evidence based decision making and Getting Research into Policy and Practice (GRIPP). In this article, we use KT as an all-encompassing term defined as 'a dynamic and iterative process that includes synthesis, dissemination, exchange and ethically sound application of knowledge to improve health, provide more effective health services and products and strengthen the health care system'[3].

### Review of available KT frameworks

Several KT frameworks have been developed and arguments for and against each of them have been raised. Some have been judged as un-realistic, like the linear model which implies that disposing of research findings will impel action [4]. The problem-solving model, which starts from identifying a problem and searching for evidence to address it, has not always been successful [4]. The incremental models, including the enlightenment and interactive models infer the notion of gradually influencing ideas among policymakers, but have been criticized for failing to make a direct link between research and its use [5,6]. In the political model, research findings are used by politicians to justify their actions, while in the tactical model, policymakers' commission research when faced with pressures. The latter 2 have been criticized for putting emphasis on the process rather than research findings [7]. The diagonal model focusing on the interactions between researchers, policymakers and lay people has been noted to be complex [4,5].

McDonald raised the issue of research being seen as a retail commodity that is relevant, easy to understand and easily accessed [8]. In practice, the contrary is often seen and relying on passive diffusion and acquisition of knowledge by policymakers is unlikely to result in changes in practice [9]. Young (2005) suggested a more comprehensive framework identifying 4 groups of factors that influence uptake of evidence by decision-makers: (a) external influences; (b) the context encompassing politics and institutions; (c) the type of evidence, its quality and how it is communicated and; (d) stakeholders, their links and their roles, including legitimacy of researchers [10]. While this presents the elements of a comprehensive framework, it still lacks a systematic approach that explains KT from the point of setting the research agenda to actual implementation.

Some frameworks have been criticized for putting emphasis on evidence, how it is disseminated and implemented with limited attention paid to the highly political and rapidly changing policy making context which is common in low income countries [11-16]. Other frameworks have proposed a range of activities along several domains without prioritization [17,18]. Oxman et al noted that little is known on how best to organize these range of activities to improve uptake of evidence in policy development [19]. Lomas and Dobrow et al's frameworks focused on the nature of the evidence, linkages between policy makers and researchers, structures for decision making and contextual issues with limited attention to the involvement of stakeholders deemed important in low income countries specifically communities and civil society [18,20]. Sauerborn et al developed a stakeholder orientation model for policy making to enhance use of health system research in health sector reform [6]. The main focus of this framework is how evidence interfaces with stakeholders with minimal attention to the context. The COHRED Working Group [21] proposed a holistic framework encompassing paying attention to the process of planning and executing research and, decision-making; having platforms for researchers and policymakers'; stakeholders identification and involvement; proving high quality and relevant research; fostering linkages between the research and policy processes and; context- the environment surrounding the research and decision making processes. This framework however does not highlight capacity requirements for the framework to be used.

There are other political science frameworks that have looked at how issues get on to the policy agenda acknowledging that there are multiple streams, research being just one of them [22,23]. These two frameworks highlight the importance of a "policy window". They use a different approach where the starting point is the policy process as opposed to evidence and one cannot make a direct link between evidence and its uptake.

Some researchers have argued that available KT models are academic and too complicated to capture the complexity of the processes involved and as such, not user-friendly for programme managers or policymakers [24]. Eastabrooks et al stated that there is no overarching KT theory and that available theories were focused on narrow areas [25]. In the case of low income countries, major shortcomings of all these models have been pointed out by Young [10]. These include the chaotic nature of policy making that may not allow enough consideration for evidence infusion, exaggerated role of donors, the problem of research supply and the role of civil society [10]. Other studies have cited challenges with donor dependence in low-income countries where undertaking research has been set as a precondition to accessing external loans [21]. Cultural differences and contexts are important, and for KT models to explain uptake of evidence, the KT theory must fit into a given context [4,25]. Few studies exist on KT in low-income countries, and inconsistencies in factors identified as influencing use of evidence have been noted in the few studies that are available [26].

Shampa highlighted the need to look at KT as a process that should be addressed right from the pre research/evidence generation stage to actual utilization of results into policy and implementation [24]. Favourable factors should be identified at the different stages and likewise, anticipated barriers should also be identified and addressed. This paper aims at contributing to this. Its main objective is to elaborate a Middle Range Theory (MRT) of KT in Uganda that can also serve as a reference for other low- and middle countries. MRTs should be understood here as defined by Merton in 1968 as "theories that lie between the minor but necessary working hypotheses (...) and the all-inclusive systematic efforts to develop a unified theory that will explain all the observed uniformities of social behavior, social organization and social change"[27]. Our preliminary MRT is built on the basis of a literature review and we further refined it through Key Informant (KI) interviews with policymakers and researchers in the health sector in Uganda. We present a range of facilitators at the different stages of the policy development and implementation process as perceived by policymakers.

## Methods

This two-step study employed qualitative approaches to examine the principal barriers and facilitating factors to KT. Step 1 involved a literature review and the identification of common themes, which were incorporated in a preliminary MRT. The latter was further refined through KI interviews with policymakers and researchers in Uganda in step 2.

### Literature review

We located relevant articles by searching PubMed, Google Scholar and Entrez for empirical papers examining translation of research into policy. We used search terms including (OR) "research", "evidence" combined with (OR) "implementation", "uptake" combined with (AND) "health policy", "low income countries". Publication bibliographies of experts in the domain were searched to identify additional relevant literature.

**Figure 1:**
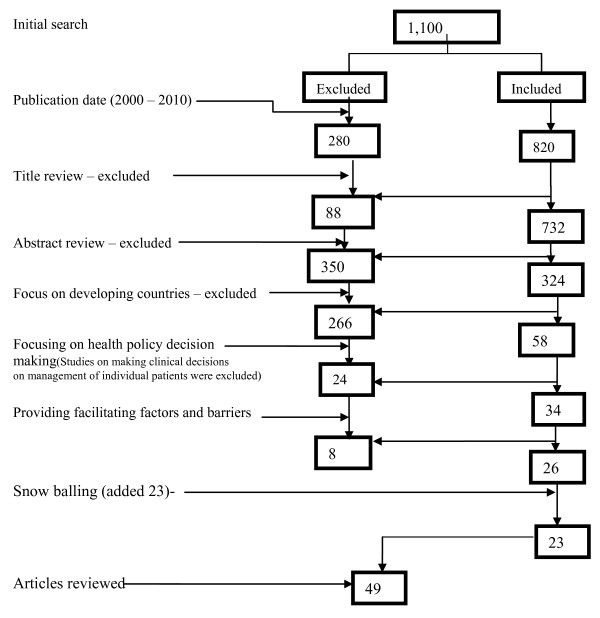
**Inclusion and exclusion criteria**.

The inclusion criteria were papers published between 2000-2010, with a focus on low income countries and on public health policy as opposed to clinical decisions and; providing facilitating factors and barriers to KT as shown in Figure 1.

Although this search was not systematic, we searched the major databases and believe the reviewed papers are a representative sample on which a plausible MRT could be based. Dixon-Woods et al state that this is warranted if the focus of the review is development of a theory rather than exhaustive summary of available literature [28].

Articles were reviewed in a two-step process. First, articles and abstracts were reviewed for inclusion criteria. Second, the remaining articles were read by the first author and vetted further by 2 of the co-authors on the basis of inclusion criteria. Articles were then coded by the first author and the reported findings grouped into facilitating factors and barriers to KT at the different stages of undertaking research, policy development and implementation in different contexts. Forty-nine articles met the inclusion criteria and were reviewed. Details of the reviewed papers are shown in Additional file 1:Table S1. The document review took place between February - October 2010. Emerging facilitating factors and barriers to KT were identified and integrated into a MRT on improving KT.

### Key informant interviews

The second stage of the study consisted of in-depth interviews with key informants (KI). Seventeen health policymakers and researchers were purposively selected on the basis of their occupation as policymakers (n = 15) or researchers (n = 2) in the area of health system development.

Information regarding the selected KI is shown in Table 1. All KIs were members of the Health Policy Advisory Committee (HPAC), the policy advisory body of the health sector. It comprises of senior government officials from the central and district level, representatives of donor agencies, the private not-for-profit (PNFP) and the private-for profit sector (PFP). The process of policy development begins with technical discussions within technical working groups comprised of government officials, donors, researchers, civil society and private health providers. Technical working groups propose options which are discussed further in HPAC and a final decision regarding which final policy option to adopt is taken.

**Table 1:** Key informant respondents

Sector		No. in HPAC	No. selected
	Ministry of Health (5)		

Public	Central level	9	4
	
	District level	1	1
	
	Researcher from School of Public Health	-	1

Private	Private not for profit (Civil society) (4)		
	
	Facility based	2	2
	
	Non facility based	2	2

	Researcher	-	1

	Private for profit	1	1

Donors	Bilateral	4	2
	
	Multilateral	3	3

**Total**			**17**

Officials in the HPAC are the most senior officers in their institutions/agencies. In the case of the PNFP and PFP organizations, they are delegated representatives of umbrella bodies. Districts are represented by officers of local government health services. Two researchers were selected on the basis of their previous work on KT and involvement in making research relevant to policy development. Interviews were conducted between November 2010-January 2011.

An interview guide was developed in line with the MRT that was drawn from the literature review. The informants were not exposed to the MRT before the interview. Questions were open ended asking for the informants' perception of barriers and facilitating factors to KT. Further probing was done to elicit responses in line with the MRT. Once the informant had given responses of perceived barriers and facilitating factors, (she) he was then asked to rank the top three in order of importance, and to indicate the reason. The interview guide was pilot tested and revised accordingly. KIs were initially contacted by email or telephone and invited to participate in the study. Informed consent was solicited prior to the interview and all interviews were conducted face-to-face by the first author. All interviews were recorded and after each interview, the interviewer made additional notes to record initial findings and impressions. The interviews were transcribed verbatim. Deductive content and thematic analysis was carried out and responses were analysed on the basis of the elements of the MRT to assess the degree of convergence and identify other emerging issues Additional file 2.

This study was approved by the Institutional Review Board of the Institute of Tropical Medicine, Antwerp (Belgium) - IRB number IRB/AC/ac/197.

## Results

In this section, we first present the summary of the findings from the literature review.

### Findings of the literature review

Facilitating factors for KT were identified from reviewed papers and categorized in 8 themes:

1. Institutional strengthening for KT

2. The pre-research phase: research priority setting

3. Research characteristics

4. Dissemination

5. Contextual issues: politics and economic considerations

6. External influences: global evidence and donor influences

7. Partnerships

8. Health System Strengthening (HSS) considerations

A summary of the commonly mentioned facilitating factors under each group is shown in Table 2. Facilitating factors under the themes 'strengthening institutional capacity for KT", research characteristics', 'dissemination', 'political context' and 'partnerships' were mentioned in more than 50% of the retained papers. We present these in more detail as they guided the development of the MRT.

**Table 2:** Summary of results (number of reviewed papers mentioning a specific facilitating factor) and themes for the MRT for KT

		Most commonly mentioned facilitating factors	Themes for the MRT for KT
	Strengthening institutional capacity for KT	- Capacity of policymakers for knowledge management (central depository, research processes, interpretation, synthesis and application)	- Capacity in knowledge management (central depository, research processes, interpretation, evidence based culture, ownership of results, synthesis and application)
		
1		-Ownership of research results by policymakers	(39)
		
		-Having policymakers with a research background and researchers skilled in policy making	
		
		- Availability of an institutionalized mechanism of getting researchers involved in policy making and policymakers involved in research	- Institutionalized mechanisms for researchers and policymakers engagements (26)
		
		-Supportive policy framework for implementing research results (guidelines, plans, monitoring frameworks)	-Supportive policy framework (7)

2	Pre-research phase	-Prioritization of research addressing policymakers information needs	-Research being part of a prioritized research agenda (21)

3	Research characteristics	- Timely, relevant, high quality and comprehensive evidence with recommendations offering policy options	-Timely provision of high quality and contextualized evidence with recommendations that are economically feasible and offering policy options
		
		- Contextualized evidence (Considering political, social, cultural, religious norms and	(40)
		
		- Credible researchers **(28)**	-Credibility of researchers (28)
		
		-Use of local researchers	-Local researchers (7)

	Dissemination	- Knowledge brokers play a role	-Use of knowledge brokers (9)
		
		- Publishing simple clear messages	
		
4		- Extensive dissemination using multiple strategies for different stakeholders	-Use of simplified language disseminated through multiple strategies that are audience tailored (35)
		
		- A dissemination plan, target audiences, dissemination activities, research products must be developed and funded	-Availability of a dissemination plan that is funded (10)
		
		Face to face interactions	-Meeting policy makers face to face (11)
		
		- Use of demand driven research networks that respond to national decision makers questions	- Use of demand driven research networks that respond to national decision makers questions (3)
		
		Providing access to internet and searchable databases	Improving internet access (6)
		
		-Presence of a champion passionate about translation of results and able to push issues to the policy table	-A champion who gets evidence to the policy table (6)

5 Context	Political	- Political stability	
		
		- A political environment that conducive to policy making and open to change	- A politically favorable environment that is open to change
		
		- Credibility of concerned government officials	(26)
		
		- Getting political involvement in research	
		
		- Presence of a policy momentum	
		
		- Availability of a political window **(7)**	-Availability of a political window (7)
	
	Economic	- Availability of funding for undertaking research, KT activities and implementation of recommendations	-Availability of funding (20)

6 External influence	Global evidence	-Support from global respected bodies like WHO	- Support from global respected bodies like WHO
		
		-Global evidence supporting local decision making	(13)
		
		- Global evidence in line with local evidence	-Global evidence supporting local evidence (10)
	
	Donor influence	-Donor influence on both the research process and policy development given donor dependence in low income countries	-Donor influence on research and policy making given their important financing role in low income countries (10)

7	Partnerships	-Mutual and trusted partnerships sustained through systematic platforms for interaction with all stakeholders right from the time of setting the research agenda to implementation of recommendations	-Mutually and respected partnerships involving communities spanning the whole process starting at setting the research agenda (35)
		
		-Specific involvement of communities	-Community involvement (7)
		
		-Use of regional networks as a way of creating interaction	-Use of regional networks as a way of creating interaction (8)
		
	HSS issues	-Monitoring system to enforce implementation of recommendations	
		
8		-Availability of guidelines to support implementation of research recommendations	
		
		-Giving appropriate financial incentives to implementers	-Capacity of the HSS to implement recommendations including provision of incentives to implementers
		
		-HSS capacity to implement recommendations in terms of HRH (numbers and skills), availability of inputs and infrastructure	(18)

### Institutional strengthening for KT in the health sector

Thirty-nine out of 49 papers reviewed showed that efforts must be made to build relevant capacity among policymakers in research processes, synthesis and application of evidence. Having policymakers with a background in research has been noted to be beneficial to KT. This contributes to ownership of research results and better uptake in policy development and implementation. Institutionalized platforms for engagement between researchers and policymakers right from setting the research agenda to policy development and implementation need to be in place for effective and continuous dialogue. This enables policymakers to appreciate the research processes and ensure their involvement in evidence generation. On the other hand, it enables researchers to appreciate the policy process, implementation challenges and to develop relevant research questions. A supportive policy framework to enable implementation of research findings is also noted to be important.

### Research characteristics

Forty out of 49 papers reviewed showed that evidence is taken up better, if it is (perceived to be) rigorous, contextualized and provided in a timely manner by credible researchers. Evidence must be comprehensive as much as possible, looking at several dimensions (for example cost implications and implementation feasibility) so as to enable decision making. If recommendations are provided, they must be feasible from an economic and implementation point of view and provide options for short-, medium- and long-term strategies. Local researchers are reported to have the advantage of engaging with policymakers for longer periods and to contextualize findings better as opposed to international researchers.

### Dissemination

Thirty-five out of 49 papers reviewed highlight the importance of effective dissemination of evidence. Messages must be simplified, tailored to the different audiences and disseminated using multiple approaches (push efforts). Personal communication and face-to-face interactions between researches and policymakers are mentioned in several papers as effective strategies. Dissemination should be planned for right at the beginning of the research processes, target audiences mapped, dissemination activities agreed and funding provided. Knowledge brokers with the capacity to package evidence in several ways have been shown to facilitate dissemination efforts. Literature however shows that these must be independent and have the capacity to manage conflict of interests among stakeholders. Improving access to internet as ways of improving dissemination was mentioned in 6 papers. Three papers mentioned the use of demand-driven research networks/rapid response units that can respond to the needs of policymakers for evidence in a timely manner. Availability of a champion, who is passionate about KT and gets issues to the attention of policymakers, was found to be an additional facilitating factor.

### Political context

Twenty-six papers stated that the political environment must be favorable and supportive to adopt and implement the generated evidence. Government stability, openness to change and dialogue provide better chances for KT. Presence of a political window of opportunity and a strong policy momentum are also mentioned as facilitating factors.

### Partnerships (exchange efforts)

Thirty-five papers showed that sustained partnerships spanning the whole process, from evidence generation to its application with all relevant stakeholders creates transparency in evidence generation. This subsequently increases the acceptability of evidence among stakeholders, which in turn facilitates KT. Oxman A. et al, however raises the question of how best to engage the different stakeholders in regards to the degree of involvement, forum for communication, method of recruitment and building their capacity for effective involvement [19].

Elements of the MRT emerging from the literature review can be summarized along the five themes as follows:

#### Institutional strengthening for KT

Adequate capacity for knowledge management and institutionalized mechanisms for researcher-policymaker interaction contribute to higher ownership and consequently, better application of evidence.

#### Research characteristics

Timely provision of high quality, contextualized evidence with feasible recommendations and policy options leads to higher adoption and implementation, especially if produced by credible, local researchers and if generated through demand-driven research networks that respond to national decision makers' priorities

#### Dissemination

Effective provision of research findings is facilitated by using knowledge brokers, audience-tailored formats, and dissemination through multiple channels. Policy champions enable evidence to reach the policy table.

#### Political context

A politically favorable environment open to change and the availability of a political window will make policymakers more receptive for evidence that has the above features.

#### Partnerships

Partnerships spanning the whole process from setting the research agenda to implementation are important and should include communities and implementing actors. Regional networks may contribute to better exchange of experience.

**Figure 2:**
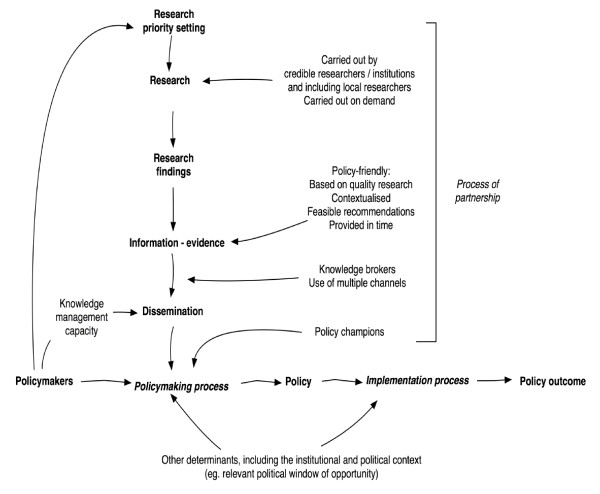
**Diagrammatic presentation of the initial MRT**.

Figure 2 shows the diagrammatic presentation of the MRT. The figure presents the different elements of the MRT, showing how the elements dealing with research link with the policymaking process.

### Further refining of the MRT through interviews

We sought to further refine the elements of the above MRT using data from KI interviews through assessing the degree of convergence or divergence and also sought to identify new emerging themes. As mentioned in the methodology section, we undertook KI interviews with officials involved in policymaking and holding senior positions in their agencies/institutions. KIs were all senior level officials 15 of whom were policymakers and 2 were researchers. Civil society respondents had been in post for at least 6 years except one who had been in post for 3 years. Ministry of health respondents had been in post for at least 6 years except one (3 years). Donor respondents had been in post for at least 6 years except two (six months and 2 years). In the case of researchers, one had been in post for 14 years and the other 2 years. The private for profit respondents had been in post for 2 years.

The interviews were analyzed along the themes of the MRT.

A summary of the interview results shows that the interviewees focused most on institutional strengthening for KT, research, dissemination, political context and partnerships (Table 3).

**Table 3:** Summary of results from KI - responses (number of respondents)

	Strengthening institutional capacity for KT	-A strong policy analysis unit within MoH (1)
		
		- Capacity in knowledge management (central depository, research processes, interpretation, evidence based culture, ownership of results, synthesis and application) (11)
		
		-Research advisory network bringing together researchers, civil society, knowledge brokers, policy makers (1)
		
		-Availability of an institutionalized mechanism/platform of getting researchers involved in policy making and policy makers involved in research (12)
		
		-We need to have an implementation and evaluation framework for KT in place (1)
		
		-Reduce the extent of bureaucracy in the policy making process (1)
	Pre-research	- Availability of a research agenda to ensure that research addresses gaps (12)

	Research characteristics	-Timely provision of high quality and contextualized evidence with recommendations that are economically feasible and offering policy options (15)
		
		-Credibility of researchers (6)
		
		-Use of local researchers (2)
		
		-Who commissions the research (1) -Separation of roles between researchers and policy makers (1)

	Dissemination	-Designated team in government to handle dissemination - knowledge brokers (1)
		
		-Simplified, well packaged and summarized messages disseminated using multiple channels tailored to targeted audiences (12)
		
		-Each research should have a communication strategy developed and funded (2)
		
		-Use of radio and also politicians can play a role in dissemination especially at community level(6)
		
		-Key role played by civil society but blocked by middle man, the government (5)
		
		-Use of electronic media (2)
		
		-Need a champion who gets to who the real policy makers (1)

Context	Political	-Favorable political environment open to change (7)
		
		- Availability of a political window especially around elections (5)
		
		-Politicians involvement in research including targeted dissemination to them (9)
	
	Economic	-Government commitment to implement recommendations from research (7)
		
		-Economically affordable recommendations (8)

External influence	Global evidence	- Interaction with WHO which gives authoritative advice (6)
		
		-Global evidence is guiding countries, if local evidence is in line, it helps (7)
	
	Donor influence	-Donors are helpful in providing funding to improve uptake of research (12)

	Partnerships	-Involvement of all relevant stakeholders throughout the process to improve trust and build interest (15)
		
		-Communities are very important (6)

	HSS	-Capacity of the HSS to implement recommendations including provision of incentives to implementers(9)

### Institutional strengthening for KT

Most respondents (11/17) highlighted the need to build the capacity of policymakers in research processes, synthesis and application. A civil society respondent mentioned that "*We need leaders who understand the evidence, this is very necessary- enlightened leaders can easily understand evidence and apply it"*.

Researchers raised the issue of an improved reading culture as a facilitating factor for effective discussion and appreciation of research results. One researcher noted that *"The reading culture is poor among the stakeholders. You cannot disseminate through drama for these highly educated people. (...) Some officials come to the workshop when they have not read the research paper. You give them some hours to read and resume the workshop. This is also not good because within that short time, they will not understand everything to contribute new ideas"*.

Most respondents (12/17) stated that a systematic dialogue and engagement with all stakeholders through institutionalized platforms was one of the ways to ensure that the set research agenda is followed, and evidence is well disseminated and implemented. This helps researchers to participate in policy dialogue and understand the system, while policy makers on the other hand appreciate the research process better. A MoH respondent remarked that "*We need to institutionalize a framework of linking policy makers, researchers and implementers. There are weaknesses within the MoH to get in place formation of a research agenda in a consultative manner. The Uganda National Health Research Organization, that should play that role, is very weak. Research undertaken is largely not commissioned. Without a formalized way of using evidence to change policy, KT will continue to be a challenge."*

Policymakers, however, stated that these platforms should include more than just policymakers and researchers. They should be broadened to include civil society.

A MoH respondent stated that the MoH should take over overall coordination of evidence generation and dissemination activities and mentioned that a policy analysis unit embedded within the MoH is best placed to coordinate the platform.

A PNFP respondent pointed out the need to reduce the bureaucratic and protracted nature of decision making and policy development and remarked that we need to "*Reduce the extent of bureaucracy within the policy making process, reduce the levels of decision making, in this way one can keep track of decisions being made at the different stages and use of evidence in decision making"*.

### Research

Virtually all respondents stated that the scientific soundness, relevance, timeliness, comprehensiveness of the evidence and feasibility of provided recommendations were important. One respondent remarked that "*Research must be credible if it is going to drive policy. There is no cause to fear if research has been done by WHO or the School of Public Health, there is no doubt of its credibility*. (MoH respondent)

The credibility of researchers was mentioned by 6 interviewees, who specified attributes like researchers of good standing, being reputable and being independent. One responded raised the issue of corruption affecting the research community and research in general: *Credibility of researchers matters also. You must get researchers who are respected, otherwise nobody will believe in the results. We, however, have a problem these days. Corruption is also affecting the research process. Some researchers undertake fieldwork and when you look at the data, you really wonder whether they actually went to the field. We are now tending to rely more on Uganda Bureau of Statistics and surveys supported by Macro international, whose quality is undoubtable. (*MoH respondent).

Two respondents mentioned that use of local researchers as a favorable factor, because they can easily contextualize research findings and engage more with policymakers. "*It is better to use local people in undertaking the research. In that case; results are more likely to be well received and used because they already take into account local context issues*" (Donor respondent).

The issue of who commissions the research was raised by a PNFP respondent, who stated that "*If research is not commissioned by MoH or WHO, it may not be taken seriously"*.

One MoH respondent mentioned the need to separate roles between researchers and policymakers. He stated that "*We should separate roles, research should be undertaken by researchers who are independent, can assess issues critically and objectively. Policymakers should then receive results, discuss them, understand them and use them in policy development"*.

### Dissemination

The majority of respondents (12/17) raised the issue of better dissemination of evidence through well packaged and adapted messages, using multiple dissemination channels that are tailored to different audiences. One PNFP respondent stated that *"All stakeholders must be part of the dissemination. Simplified messages in several forms, policy briefs, report summaries, can be extensively circulated. We need to take advantage of existing fora; people should present their research results in these fora."*

One respondent mentioned the importance of face-to-face dissemination, especially to senior people in their offices, while 6 mentioned use of radio and politicians as the best way to reach the community. One donor respondent pointed out the need for multiple channels: *"We need to use various opportunities, formal and informal to disseminate research results. Radios can be used but must be repetitive short massages. Most important is to use various channels. One misses this one, but catches another channel."*

Four PNFP and 1 donor respondents mentioned the significant role that civil society can play in disseminating evidence, although they again noted the challenges that need to be addressed for this to be effective. One PNFP respondent stated that "*A well informed and knowledgeable civil society can play a role in dissemination, because we have roots in the community, advocacy, and community mobilization. We can put pressure on policymakers to implement evidence, but we are largely left out. The challenge is that we have to go through that middleman, the government. The government to some extent blocks civil society by putting restrictions on "who" should undertake dissemination*".

Two respondents mentioned use of electronic media although they acknowledged its limited accessibility and thus the need to assess its usefulness. One researcher remarked: "*Media and IT are not accessible by all. A lot of research is disseminated through electronic media, but some areas have no such infrastructures, same do not have e-mail, we need to evaluate its usefulness"*

One PNFP respondent mentioned the creation of a team within government structures to be charged with the responsibility of disseminating evidence and remarked that "*There must be a team in government unit who handle dissemination other than leaving to individual programmes and researchers. They should move quickly and reach out to the researchers and make sure that available evidence informs policy quickly"*.

Four respondents stated that dissemination is a process that should be planned for at the very beginning of the research process, and that a communication strategy needs to be developed and funded.

One donor pointed out the importance of having a champion within the MoH who links up with higher-level officials who actually make policy decisions. Such champions are able to go and see the different officers and engage in face-to-face dissemination. The respondent stated that "*It helps a lot to have a champion within MoH who links up with higher officials, who actually make policy decisions. He can spend more time with senior officials and ensure that results are shared in important fora"*.

### Political context

Seven respondents mentioned the need for a favorable political environment that is open to dialogue and a political window that presents an opportunity for change. A PNFP respondent stated that "*The best time to disseminate results is when there is a political window like at the time of elections, then politicians pick it to their advantage. But if the research result is affecting the incumbent government, it may not be a good time for you to disseminate such a result"*.

Specific mention by 9 respondents was made of getting politicians' involvement in research and among suggested strategies is targeted dissemination as stated by a MoH respondent that "*Politicians are very important, they should be a targeted group because they are in the community, and there are top policymakers so they should be given high consideration"*.

### Partnerships

Almost all respondents cited the importance of involving all relevant stakeholders throughout the process, right from setting a research agenda to policy development and implementation. Six respondents mentioned the need to involve communities who are often neglected. One PNFP respondent stated that *"The community is not involved at all. It's only policymakers and researchers, the community is neglected, yet they are the main beneficiaries who know their problems too well"*.

### Ranking of facilitating factors by key informants

We asked respondents to rank the facilitating factors in order of importance (Table 4). The top 3 facilitating factors were 'strengthening institutional capacity for KT' (12 respondents), 'research characteristics' (9 respondents) and 'partnerships' (9 respondents). No respondent ranked political context among the top three facilitating factors. MoH and PNFP respondents emphasized the importance of institutional strengthening for KT. Donors emphasized research characteristics, while researchers emphasized partnerships.

**Table 4:** Number of respondents ranking facilitating factors among the top 3

	No. of respondents ranking as:	Institutional strengthening	Research	Partnerships	Dissemination	Political context
MoH	1	2	0	1	1	0
	
	2	2	0	2	0	0
	
	3	0	1	1	0	0

Donors	1	1	1	0	0	0
	
	2	1	1	0	3	0
	
	3	0	3	0	0	0

PNFP	1	2	0	0	0	0
	
	2	1	1	1	1	0
	
	3	1	0	1	2	0

PFP	1	1	0	0	0	0
	
	2	0	0	1	0	0
	
	3	0	0	0	0	0

Researchers	1	0	0	2	0	0
	
	2	0	1	0	0	0
	
	3	1	1	0		

No. ranking a facilitating factor among the top 3		12	9	9	7	0

### Refining the MRT

Taking into account what is stated as most important by policymakers in Uganda, we refined the initial MRT that was based on the literature review under three themes as follows.

#### Institutional strengthening for KT

- Institutionalized platforms for engagement between researchers and policymakers including civil society

- Mainstreamed mechanisms (within MoH) to coordinate evidence generation synthesis and dissemination

- Build capacity of policymakers in knowledge management (central depository, research processes, interpretation, evidence based culture, ownership of results, synthesis and application

- Reduced bureaucracy in policy making

*Expected outcome*: higher ownership and better application of evidence

#### Research characteristics

- Timely provision of high quality and contextualized evidence with recommendations that are economically feasible and offering policy options

- Credibility of researchers

- Use of local researchers

- Separation of roles between researchers and policymakers

*Expected outcome*: higher adoption and implementation

#### Partnerships

- Involvement of all relevant stakeholders throughout the process to improve trust and build interest

- Community involvement in evidence generation and KT

*Expected outcome*: higher adoption and implementation

**Figure 3:**
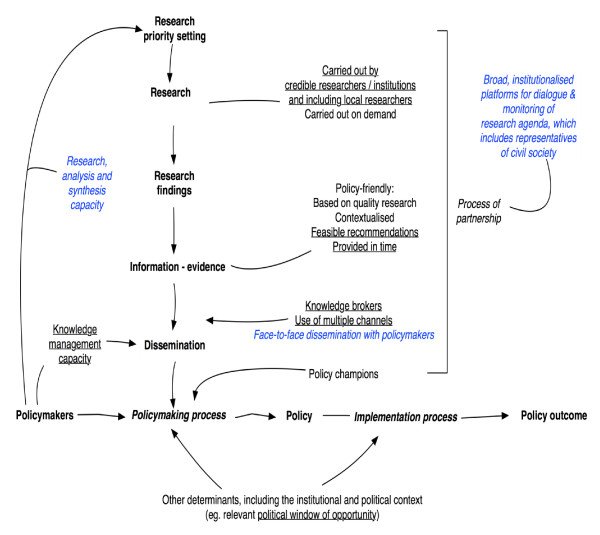
**Diagrammatic presentation of the refined MRT**.

Figure 3 presents the modified MRT with the elements confirmed by the interviews now underlined and new elements in blue italic type. This shows that policymakers, researchers and other actors involved in policymaking in the health sector in Uganda believe that particular attributes of the research and the dissemination process influence the uptake of research and knowledge into decisions on health policies, but that this requires sound institutional capacities. They believe that several factors can facilitate such uptake: the presence of political windows of opportunity and the involvement of knowledge brokers and policy champions.

## Discussion

Strengthening institutional capacity for KT, research characteristics and partnerships were ranked among the top three facilitating factors and these are discussed further. The analysis of the key informant interviews indicated that there was a convergence between the findings of the literature review, available frameworks and the perceptions of the key informants in many areas, but there were also divergences. Some other issues emerged in regard to what policymakers felt were favorable factors to KT.

Concerning institutional strengthening, this study shows that policymakers favor broadening the institutional platforms for KT in the health sector beyond researchers and policymakers to include civil society. This is contrary to several of available KT frameworks where emphasis has been placed on having platforms between policy makers and researchers [6,14,17,18]. This may be because of the increasing role currently played by civil society in research, priority setting and KT in low income countries and, in that case their inclusion becomes important [29]. Sanders et al, for instance, stated the important role CSOs play in supporting innovations and making research relevant to communities [30]. Evidence shows that the role of civil society organizations (CSOs), the media and pressure groups in KT largely remains unexplored [4]. This study has also showed there are tensions between the government and civil society when it comes to advocating for uptake of evidence and these need to be addressed if civil society is to play its role in KT effectively. Lomas, in his framework of connecting research and policy, raised the issue of having a voice, conduct within an institution and organizational culture as an important factor for uptake of evidence [18]. Similar findings have been reported in other low income countries where successful involvement of civil societies in KT and policy processes was hampered by suspicions between the government and civil society [10]. The role of civil society in evidence dissemination needs further exploration through research as highlighted in previous studies [29,30].

Policymakers expressed preference for stronger structures within MoH to undertake overall coordination of research processes and KT. This is in line with ongoing efforts of strengthening government stewardship in research processes in low income countries [31]. Such structures are more likely to improve access to relevant audiences, have insights in topical policy issues and broader health sector concerns enabling them to engage more effectively in KT. Similarly, Varkevisser et al in their study on research in action for Southern African countries emphasized strengthening national structures to coordinate health systems research [32]. As a point of caution however, one needs to safeguard against government attempting to assume overall control over the KT process as has been highlighted by a civil society respondent in this study. Lavis et al also noted that government support units that were more focused on public health, involved target users more and had more informal relationships with policymakers [33]. Contrary to this however, Boween et al, in their framework on evidence-informed policy and practice; proposed use of external structures like professional bodies and networks [11]. Lavis et al also documented successful experiences with use of structures outside government although these were in the area of clinical practice guidelines and health technology assessment [33,34]. This may imply that, where polices are of a public health nature as opposed to health care/clinical, mainstreamed structures may do better that structures outside governments.

Respondents stated that required capacity must be built among policy makers in research processes, interpretation, synthesis and application of evidence. They must have an evidence based culture if they are to own and use evidence. Several KT frameworks have emphasized the importance of capacity among policy makers in knowledge management as important for uptake of evidence [11,12,15,17,20,21]. We also found that policymakers believe that besides a body dealing with research coordination, there is also a need for a designated team within MoH charged with the responsibility of synthesizing and disseminating evidence. They argued that it may enhance ownership of generated evidence and commitment to implementation of recommendations by the government. The use of demand driven research networks responding to policymakers' evidence needs, as identified in the literature, was not much mentioned as a facilitating factor by policymakers. Reasons for this could be twofold: either the interviewees believe that they indeed play no role or it could be that policymakers' lack of experience of using these networks hampers their appreciation of its usefulness. We note that although the Regional East African Community Health (REACH)-Policy initiative was put in place by ministries of health of the three East African Countries (including Uganda) in 2005 to support use of evidence in policy development, no respondent made mention of this initiative. A possible reason could be the high turnover of government officials in that, the team that committed to the initiative may no longer be in office. Indeed the Ministry of health has had important changes in ministers and other personnel in the previous years. Stability in researchers' personal and organizational contacts has been noted to be critical and the high turnover of national health authorities cited as one the challenges to uptake of evidence in policy development [32,35]. Another reason could be poor communication within government structures in that a commitment that was made at a ministerial level was inadequately communicated to other policy makers. It could also be that our respondents have not made use of this initiative.

Our study has shown that some policy makers believe that reduced bureaucracy in the policy development process, as pointed out by civil society respondents, is favorable for KT. Indeed evidence has shown that government bureaucracy impacts negatively on uptake of evidence in policy development. Some of the available frameworks have raised the issue of organizational bureaucracy as an important factor in determining uptake of evidence, the extent to which an organization is open to debate on research findings [11,12,20,21]. Similarly Sevene et al found that communication failure within government bureaucratic processes obstructed delivery of cost effective interventions [36]. Mubyazi and Gonzalez-Block noted the challenges of keeping track of use of evidence in protracted policy development processes [37]. A lengthened and bureaucratic policy development process poses a risk of delays and calling for fresh evidence as issues change.

Under research characteristics, respondents mentioned the need for provision of relevant, high quality, contextualized evidence with feasible recommendations which is also highlighted in several KT frameworks [11-13,16,20]. This study has brought to the forefront that policymakers prefer separation of the roles of research undertaking and policy development. Contrary to this, Sauerborn et al in their stakeholder orientation model for policy making to enhance use of research, raised the need for use of researchers inside MoH as one of the options for creating an appropriate institutional framework [6]. Our findings are however, in line with other studies. For example, Young documented successful experiences in a few African countries, where commissioned research undertaken by independent think tanks within the countries informed policy development [10]. However, such an approach may not work in cases of action research where interventions are piloted in order to learn lessons to guide large-scale implementation or in the case of operational research addressing management problems faced by health managers at the work place. Indeed Varkevisser et al found that researchers within Ministries of health were able to undertake research on management problems they faced in their working environment and successfully implemented recommendations [32]. This implies that, a strict separation of roles as highlighted by our respondents must be assessed on a case by case basis. Policy makers expressed preference for the use of local researchers. This may be beneficial in several ways; they can engage with policy makers for a longer period of time and can contextualize evidence better compared to international researcher. The challenge noted is however the weak capacity among local researchers in low income countries to produce rigorous quality evidence [38,39]. In this case, it may be wise to explore partnerships between local and international researcher teams.

Under the theme of partnerships, several frameworks have similarly highlighted the importance of sustained partnerships in evidence generation, dissemination and implementation [11-13,18,20,21]. In our study, policymakers put a lot of emphasis on community involvement in addition. The role of communities in policy making and enforcing accountability has increased in the recent past more especially in low income countries [30]. Some KT framework have also raised the importance of community collaboration and engagement [12,21] However, Deslie et al noted that communities must be empowered in terms of skills enhancement and information sharing in order to play an effective role [29]. It should be noted that this remains often a discourse, as in Uganda, the communities are little involved in research priority setting.

The use of regional networks and professional bodies, which are stated in the literature as important, were not seen as such by policymakers in this study. Similar experiences regarding weaknesses of professional bodies to ensure uptake of evidence have been reported in other African countries. In Mozambique for example, although obstetricians participated in development of guidelines on management of eclampsia and incorporated available evidence, they later reported that they had no authority to ensure implementation of guidelines [36]. There may be different reasons for this. In Uganda, professional bodies are relatively weakly developed and have not engaged in policy development in a systematic manner. Regional networks only engage with a few policymakers and only occasionally. On the other hand, it may have to do with "power centers" in that; professional bodies and regional networks have no authority in the policy process.

In regards to the political context, policymakers did not raise the issue of political stability and credibility of government officials as important for KT. This however contradicts what has been documented in other African countries where the dialogue between researchers and policymakers was hampered by political circumstances and, un-favorable research results suppressed by political powers [32]. Possible reasons could be that our respondents are used to the high turn-over of government officials, which commonly happens in low income countries and that they may have ceased to see it as a key issue. Alternatively, policymakers may not have a full appreciation of what elements of the political context offer better opportunities for KT, and how they could deal with this. Young highlighted the need to better understand political processes in low income countries in order to improve KT [10].

Not all facilitating factors carry an equal weight in terms of influence and furthermore, their relative weight is likely to vary depending on the type of evidence and policy in question. This study showed that our interviewees ranked elements under institutional strengthening, research characteristics and partnerships as most important. Our findings are reflected by Ssengooba et al, who showed that institutional mechanisms and use of shared platforms involving all relevant stakeholders, provision of comprehensive evidence and building of coalitions of stakeholders were key for uptake of evidence in PMTCT programmes [40].

The specificity of our MRT is that it is embedded in the local political, systems and policy making context and prioritizes areas deemed important by policymakers and researchers in the country. It raises preference for use of mainstreamed structures within MoH which were thought important for sustained dialogue, ownership and institutional memory and; separation of roles between researchers and policymakers. These are less emphasized in previous frameworks. Although our MRT is developed in the context of Uganda, we believe it can serve as reference for studies in other low-income countries with contexts similar to that of Uganda. The refined facilitating factors presented in this paper were collected from respondents without a specific reference to a given research project and policy outcome. The extent to which they are valid will need to be tested on specific case studies. This withstanding, our refined MRT has proposed activities under 3 elements namely institutional strengthening for KT, research characteristics and partnerships that are within the capacity of ministries of health to undertake.

### Strengths and weaknesses of the study

The main strength of this study is the two step process that we have used to build the MRT. The refining of the preliminary MRT based on a literature review, through KI interviews with policymakers, takes into consideration the contextual issues that have been raised as a concern for KT. Among the limitation though is that factors presented in this paper were collected from respondents without a specific reference to a given research project and policy outcome. People's views may differ given the level of interest they may have in a given policy. In this study, we did not use the Delphi method to distil KI judgments and reach theoretical saturation. While that may be beneficial, we note that this approach was not appropriate given the fact that we did not refer to a specific piece of evidence and policy with relevant stakeholders mapped. The Ugandan political context may have influenced views of respondents given the fact that the country is in a multiparty system and the current government has been in power for over 23 years. The views of respondents in opposition may differ from those in the ruling party. We however did not look at the political affiliation of respondents. Although this search was not systematic, we searched the major databases and believe the reviewed papers are a representative sample on which a plausible MRT could be based.

## Conclusions

This study developed an initial MRT on knowledge transfer in policymaking in the health sector on the basis of a literature review and further refined this initial theory through in-depth interviews with Ugandan policymakers and researchers. We found that policymakers and researchers perceived a strong institutional capacity for KT, the existence of research partnerships and the characteristics of the actual research as the most important facilitating factors for improving KT. These empirical findings allowed us to refine and enrich the initial MRT accordingly. Our theory is not intended to be prescriptive, but to provide a systematic account of facilitating factors that increase chances of KT in Uganda, which can serve as a starting point for similar studies in other low-income countries.

## Abbreviations

HSS: Health systems; IT: Information Technology; KI: Key informants; KT: Knowledge Translation; MoH: Ministry of Health; MRT: Middle Range Theory; PNFP: Private not for profit; WHO: World Health Organisation.

## Competing interests

The authors declare that they have no competing interests.

## Authors' contributions

JNO contributed to the conception and design of the study, data collection, analysis, interpretation and led the drafting of the manuscript. DKM participated in data analysis, interpretation and drafting of the manuscript. BM contributed to interpretation of data and drafting of the manuscript. FS participated in conception and design of the study, data analysis, interpretation and drafting of the manuscript. JM participated in the conception and design of the study, data analysis and interpretation and drafting of the manuscript. BC contributed to the conception and design of the study, data analysis, interpretation and drafting of the manuscript. All authors read and approved the final manuscript.

## Pre-publication history

The pre-publication history for this paper can be accessed here:

http://www.biomedcentral.com/1471-2458/12/109/prepub

## Supplementary Material

Additional file 1 **Table S1**. details of publications reviewed. Click here for file

Additional file 2 **Interview guide**. Click here for file

## References

[B1] Cordero C Delino R Jeyaseelan L Lansang MA Lozano JM Kumar S Moreno S Pietersen M Quirino J Thamlikitkul V Funding agencies in low- and middle-income countries: support for knowledge translation Bull World Health Organ 2008 86 7 524 534 10.2471/BLT.07.040386 PMC2647493 18670664

[B2] Syed SB Hyder AA Bloom G Sundaram S Bhuiya A Zhenzhong Z Kanjilal B Oladepo O Pariyo G Peters DH Exploring evidence-policy linkages in health research plans: a case study from six countries Health Res Policy Syst 2008 6 4 10.1186/1478-4505-6-4 PMC2329631 18331651

[B3] Graham ID Tetroe JM Getting evidence into policy and practice: perspective of a health research funder J Can Acad Child Adolesc Psychiatry 2009 18 1 46 50 PMC2651211 19270848

[B4] Armstrong R Waters E Roberts H Oliver S Popay J The role and theoretical evolution of knowledge translation and exchange in public health J Public Health 2006 28 4 384 389 10.1093/pubmed/fdl072 17082462

[B5] Aaserud M Lewin S Innvaer S Paulsen EJ Dahlgren AT Trommald M Duley L Zwarenstein M Oxman AD Translating research into policy and practice in developing countries: a case study of magnesium sulphate for pre-eclampsia BMC Health Serv Res 2005 5 68 10.1186/1472-6963-5-68 PMC1298297 16262902

[B6] Sauerborn R Nitayarumphong S Gerhardus A Strategies to enhance the use of health systems research for health sector reform Trop Med Int Health 1999 4 12 827 835 10.1046/j.1365-3156.1999.00497.x 10632991

[B7] Elliott H Popay J How are policy makers using evidence? Models of research utilisation and local NHS policy making J Epidemiol Community Health 2000 54 6 461 468 10.1136/jech.54.6.461 PMC1731692 10818123

[B8] McDonald PW Viehbeck S From evidence-based practice making to practice-based evidence making: creating communities of (research) and practice Health Promot Pract 2007 8 2 140 144 10.1177/1524839906298494 17384405

[B9] Davis DA Thomson MA Oxman AD Haynes RB Changing physician performance. A systematic review of the effect of continuing medical education strategies Jama 1995 274 9 700 705 10.1001/jama.274.9.700 7650822

[B10] Young J Research, policy and practice: why developing countries are different J Int Dev 2005 17 727 734

[B11] Bowen S Zwi AB Pathways to "evidence-informed" policy and practice: a framework for action PLoS Med 2005 2 7 e166 10.1371/journal.pmed.0020166 PMC1140676 15913387

[B12] Dobbins M Ciliska D Cockerill R Barnsley J DiCenso A A framework for the dissemination and utilization of research for health-care policy and practice Online J Knowl Synth Nurs 2002 9 7 12439759

[B13] Graham ID Logan J Harrison MB Straus SE Tetroe J Caswell W Robinson N Lost in knowledge translation: time for a map? J Contin Educ Health Prof 2006 26 1 13 24 10.1002/chp.47 16557505

[B14] Jacobson N Butterill D Goering P Development of a framework for knowledge translation: understanding user context J Health Serv Res Policy 2003 8 2 94 99 10.1258/135581903321466067 12820671

[B15] Lavis JN Robertson D Woodside JM McLeod CB Abelson J How can research organizations more effectively transfer research knowledge to decision makers? Milbank Q 2003 81 2 221 248 10.1111/1468-0009.t01-1-00052 PMC2690219 12841049

[B16] Nutley S Walter I Davies H From knowing to doing: a framework for understanding the evidence-into-practice agenda Evaluation 2003 9 125 148

[B17] Lavis JN Lomas J Hamid M Sewankambo NK Assessing country-level efforts to link research to action Bull World Health Organ 2006 84 8 620 628 10.2471/blt.06.030312 PMC2627430 16917649

[B18] Lomas J Connecting research and policy Isuma: Can J Policy Res 2000 1 1 140 144

[B19] Oxman AD Vandvik PO Lavis JN Fretheim A Lewin S SUPPORT Tools for evidence-informed health Policymaking (STP) 2: Improving how your organisation supports the use of research evidence to inform policymaking Health Res Policy Syst 2009 7 (Suppl 1):S2 10.1186/1478-4505-7-S1-S2 PMC3271829 20018109

[B20] Dobrow MJ Goel V Lemieux-Charles L Black NA The impact of context on evidence utilization: a framework for expert groups developing health policy recommendations Soc Sci Med 2006 63 7 1811 1824 10.1016/j.socscimed.2006.04.020 16764980

[B21] COHRED Lessons in Research to Action and Policy - Case studies from seven countries. Geneva: The Council of Health Research and Development working group on Research to Action and Policy (COHRED) 2000

[B22] Kingdon J Agendas, alternatives and public policie 1995 2 New York: HarperCollins College Publishers

[B23] Lavis JN Ross SE Hurley JE Hohenadel JM Stoddart GL Woodward CA Abelson J Examining the role of health services research in public policymaking Milbank Q 2002 80 1 125 154 10.1111/1468-0009.00005 PMC2690103 11933791

[B24] Shampa N Case Studies: Getting Research into Policy and Practice (GRIPP) 2007 London: John Snow International, Europe

[B25] Estabrooks CA Thompson DS Lovely JJ Hofmeyer A A guide to knowledge translation theory J Contin Educ Health Prof 2006 26 1 25 36 10.1002/chp.48 16557511

[B26] Daniels K Lewin S Translating research into maternal health care policy: a qualitative case study of the use of evidence in policies for the treatment of eclampsia and pre-eclampsia in South Africa Health Res Policy Syst 2008 6 12 10.1186/1478-4505-6-12 PMC2645395 19091083

[B27] Merton RK Social Theory and Social Structure 1968 New York: Free Press

[B28] Dixon-Woods M Cavers D Agarwal S Annandale E Arthur A Harvey J Hsu R Katbamna S Olsen R Smith L Conducting a critical interpretive synthesis of the literature on access to healthcare by vulnerable groups BMC Med Res Methodol 2006 6 35 10.1186/1471-2288-6-35 PMC1559637 16872487

[B29] Delisle H Roberts JH Munro M Jones L Gyorkos TW The role of NGOs in global health research for development Health Res Policy Syst 2005 3 1 3 10.1186/1478-4505-3-3 PMC554095 15723694

[B30] Sanders D Labonte R Baum F Chopra M Making research matter: a civil society perspective on health research Bull World Health Organ 2004 82 10 757 763 PMC2623042 15643797

[B31] WHO WHO WHO's role and responsibilities in health research WHA6321 2010 63.21 Geneva: WHO

[B32] Varkevisser CM Mwaluko GM Le Grand A Research in action: the training approach of the Joint Health Systems Research Project for the Southern African Region Health Policy Plan 2001 16 3 281 291 10.1093/heapol/16.3.281 11527869

[B33] Lavis JN Paulsen EJ Oxman AD Moynihan R Evidence-informed health policy 2 - survey of organizations that support the use of research evidence Implement Sci 2008 3 54 10.1186/1748-5908-3-54 PMC2646748 19091108

[B34] Lavis JN Oxman AD Moynihan R Paulsen EJ Evidence-informed health policy 1 - synthesis of findings from a multi-method study of organizations that support the use of research evidence Implement Sci 2008 3 53 10.1186/1748-5908-3-53 PMC2621242 19091107

[B35] Lavis JN Guindon GE Cameron D Boupha B Dejman M Osei EJ Sadana R Bridging the gaps between research, policy and practice in low- and middle-income countries: a survey of researchers Can Med Assoc J 2010 182 9 E350 E361 10.1503/cmaj.081164 PMC2882466 20439449

[B36] Sevene E Lewin S Mariano A Woelk G Oxman AD Matinhure S Cliff J Fernandes B Daniels K System and market failures: the unavailability of magnesium sulphate for the treatment of eclampsia and pre-eclampsia in Mozambique and Zimbabwe BMJ 2005 331 7519 765 769 10.1136/bmj.331.7519.765 PMC1239984 16195297

[B37] Mubyazi GM Gonzalez-Block MA Research influence on antimalarial drug policy change in Tanzania: case study of replacing chloroquine with sulfadoxine-pyrimethamine as the first-line drug Malar J 2005 4 51 10.1186/1475-2875-4-51 PMC1277846 16242017

[B38] Hennink M Stephenson R Using research to inform health policy: barriers and strategies in developing countries J Health Commun 2005 10 2 163 180 10.1080/10810730590915128 15804906

[B39] Santesso N Tugwell P Knowledge translation in developing countries J Contin Educ Health Prof 2006 26 1 87 96 10.1002/chp.55 16557514

[B40] Ssengooba F Atuyambe L Kiwanuka SN Puvanachandra P Glass N Hyder AA Research translation to inform national health policies: learning from multiple perspectives in Uganda BMC Int Health Hum Rights 2011 11 Suppl 1 S13 10.1186/1472-698X-11-S1-S13 PMC3059472 21411000

