# Supplementary material for: Research, evidence and policymaking: the perspectives of policy actors on improving uptake of evidence in health policy development and implementation in Uganda
Source: BMC Public Health. 2012 Feb 9;12:109. doi: 10.1186/1471-2458-12-109 (PMC3305540; doi:10.1186/1471-2458-12-109)
Supplement: Additional file 1 — Table S1. details of publications reviewed. [file 1471-2458-12-109-S1.DOCX]

**Appendix 1:**

**Table 1: details of publications reviewed:**

| **No.** | **Author** | **Article** | **Year of publication** | **Objective** |
| --- | --- | --- | --- | --- |
| 1 | Stephen Hanney, Miguel A. Gonzalez-Block | Evidence-informed health policy: are we beginning to get there at last? Editorial; Health research policy and systems, 2009, 7:30 | 2009 | Editorial review highlight the importance of KT |
| 2 | Karen Daniels, Lewin Simon and The Practihc Policy Group | Translating research into maternal health care policy: a qualitative case study of the use of evidence in policies for the treatment of eclampsia and pre- eclampsia in South Africa; Health research policy and systems 2008, 6:12 | 2008 | To explore how research information, in particular findings from randomized controlled trials and systematic reviews, informed policy making and clinical guidelines development for the use of magnesium sulphate in treatment of eclampsia and pre-eclamsia in South Africa |
| 3 | Uriyoan Colon-Ramos, Lindsay A.C, Monge-Rojas R., Greaney M. et al; | Translation research into action: a case study of trans fatty acid research and nutrition policy in Costa Rica; 2007, Health policy and planning, 22: 363-374 | 2007 | To identify factors that may impede or promote the process of translation of scientific evidence about trans fatty acid into policy in the specific context of Costa Rica |
| 4 | Andy Haines, Shyama Kuruvilla; Matthias Borchert; | Bridging the implementation gap between knowledge and action for health; Bulletin of the World Health Organization \| October 2004, 82 (10) | 2004 | To provide an overview of the effective approaches used to encourage the uptake of research findings for three main groups: policy-makers, the public and health-service providers outlining additional challenges to implementation in low income countries |
| 5 | H.A Williams; D. Durrheim R. Shretta | The process of changing national malaria treatment policy: lessons from country-level studies; 2004*Health policy and Planning* vol. 19, no. 6: pp. 356–370, | 2004 | To provides important lessons about decision-making, the policy cycle and complex policy environment, while specifically identifying strategies successfully employed to facilitate policy-making and implementation. |
| 6 | Monique Hennink & Stephenson Rob; | Using research to inform health policy: barriers and strategies in developing countries; 2005, Journal of health communication, 10:163-180 | 2005 | To examine the dissemination and uptake of health research into policy and program delivery in four developing countries |
| 7 | Stephen Hanney, Miguel A. Gonzalez-Block, Buxton Martin J | The utilization of health research in policy making: concepts examples and methods assessment; 2003; Health Research Policy Systems 1:2 | 2003 | Exploring the interfaces, between researchers and policy-makers. The role of policy- makers as receivers, or receptors, of research is examined along with the accompanying institutional arrangements. Among the case studies used is health financing reforms in Zambia and South Africa |
| 8 | Crewe Emma, Young John; | Bridging research and policy: context, evidence and links; 2002; working paper 173; overseas development institute | 2002 | To increase understanding of linkages between development research, policy and practice and develop simple tools for researchers and policymakers to promote evidence-based policy that contributes to poverty reduction, alleviation of suffering or the saving of lives. |
| 9 | Nancy Santesso & Tugwell P | Knowledge translation in developing countries, 2006, Journal of continuing education in the health profession | 2006 | Testing the Ottawa model of research use in developing countries |
| 10 | Rebecca Armstrong; Waters E, Roberts H, Oliver S, Popay J | The role of theoretical evolution; 2006; J Public Health; 28(4):384-9. | 2006 | To outline some of the key concepts and debates in KT and exchange and highlighting some of the investigative work being conducted to explore KT strategies and their effectiveness. It concludes by outlining some frameworks to support public health action in KT. |
| 11 | National centre for the dissemination of disability research | Focus: Technical brief No.14 : Over view of international literature on KT – a publication of the National centre for the dissemination of disability research' 2006 | 2006 | To provide additional insight on KT from an international perspective |
| 12 | Syed SB, Hyder A., Bloom G, Sundaram S, et al | Innovation for Equity; Exploring evidence-policy linkages in health research plans: A case study from six countries; Health Research Policy and Systems; 2008, 6:4 | 2008 | Exploring a research to policy interface during proposal development in six low income countries |
| 13 | Hyder A.A, Bloom G, Melissa Leach M., Syed B. S, Peters D.H; & Future Health Systems: Innovations for Equity | Exploring health systems research and its influence on policy processes in low income countries. *BMC Public Health* 2007, **7:**309doi:10.1186/1471-2458-7-309 | 2007 | Analysis of the relationship between research (evidence) and policy making, especially their impact on the poor in six low income countries. |
| 14 | Tikki Pang; | Evidence to action in the developing world: what evidence is needed? 2007; Bulletin of the WHO | 2007 | Editorial review |
| 15 | John Young; | Research policy and practice: why developing countries are different; 2005, Journal of international development, 17, 727-734 | 2005 | To review existing literature and propose an analytical framework with four key arenas: external influences, political context, evidence and links. |
| 16 | Cynthia Cordero, Grimshaw, Tugwell et al; | Funding agencies in low-and middle-income countries: support for knowledge translation; 2008, Bulletin of WHO 86 (7): 524 - 534 | 2008 | To describe how selected health research funding agencies active in low- and middle-income countries promote the translation of their funded research into policy and practice. |
| 17 | Theobald S, Taegtmeyer M, Squire SB; Crichton J. et al: | Towards building equitable health systems in sub Saharan Africa: Lessons from case studies on operational research; 2009; Health research policy and systems 7:26 | 2009 | To use a case study approach to analyze how and why different operational research projects in Africa have contributed to health systems strengthening and promoted equity in health service provision |
| 18 | Bjorn Melgaard | From research to action – a bridge to be crossed. Editorial, WHO Bulletin; October 2004, 82 (10) | 2004 | Editorial review |
| 19 | Mubyazi GM, Gonzalez-Block MA | Research influence on antimalarial drug policy change in Tanzania: case study of replacing chloroquine with sulfadoxine-pyrimethamine as the first-line drug. Malaria J. 2005 Oct 20;4:51 | 2005 | To describe the (a) role of researchers in producing evidence that influenced the Tanzanian government replace chloroquine (CQ) with sulfadoxine-pyrimethamine (SP) as the first-line drug and the challenges faced in convincing policy-makers, general practitioners, pharmaceutical industry and the general public on the need for change (b) challenges ahead before a new drug combination treatment policy is introduced in Tanzania. |
| 20 | Aaserud. M, Lewin S., Innvaer S, Paulsen J.E, Dahlgren T. A. et al | Translating research into policy and practice in developing countries: a case study of magnesium sulphate for pre-eclampsia; BMC Health Services Research 2005, 5:68 doi:10.1186/1472-6963-5-68 | 2005 | To examined factors that might hinder or facilitate the translation of the results of the Magpie Trial into appropriate policies and practice. |
| 21 | John Walley, M Amir Khan, Sayed Karam Shah, Sophie Witter & Xiaolin Wei | How to get research into practice: first get practice into research; Editorial Bulletin of the World Health Organization \| June 2007, 85 (6) | 2007 | Editorial review |
| 22 | Kammen Van J. Don de Savingy & Sewankambo N, | Using knowledge brokering to promote evidence-based policy-making: the need for support structures; Bulleting of the World Health Organization 2006, 84 (8): 608 - 612 | 2006 | Describing experiences with knowledge brokering approach and discussing potential of this approach to assist health policy development in low income countries |
| 23 | Brambila C., Ottolenghi E., Marin C., & Betrand J.T; | Getting results used: evidence from reproductive health programmatic research in Guatemala; 2007; Health policy and planning:22:234-245 | 2007 | Exploring theoretical perspectives on the relationship between research, programme improvement and policy making |
| 24 | Tugwell P. Robinson V.. Grimshaw, J., Santesso N., | Systematic reviews and knowledge translation, 2006, Bulleting of the World Health Organization 84 (8); 643 - 651 | 2006 | Presenting a cascade of steps to assess and priorities barriers and thus choose effective KT interventions that are tailored for relevant audiences, as well as the evaluation, monitoring and sharing of these strategies - looking at ITNs and Immunization in Africa |
| 25 | Lavis J. N. Lomas J. Hamid M. & Sewankambo N. | Assessing country level efforts to link research to action; 2006, Bulletin of the World Health Organization 84; 8: 620-628 | 2006 | To develop a framework for assessing country level efforts to link research to action with implications for developing countries |
| 26 | Chunharas S., 2006, | An interactive integrated approach to translating knowledge and building a “learning organization” in health services management. 2006; Bulletin of the World Health Organization; 84: 8: 652 - 654 | 2006 | Proposing a basic approach to ensuring that knowledge from research studies is translated for use in health services management in order to build a "learning organization" |
| 27 | Por Ir, Bigdeli, M. Meesen B., Van Damme V | Translating knowledge into policy and action to promote health equity: the Health Equity Fund policy process in Cambodia 2000 – 2008; Health Policy. 2010 Aug;96(3):200-9. | 2010 | To understand how knowledge is used to inform policy on Health Equity funds in Cambodia and to draw lessons for translating knowledge onto health policies that promote equity |
| 28 | Woelk G, Daniels K, Cliff J, Lewin S, Sevene E. et al | Translating research into policy: lessons learned from eclampsia treatment and malaria control in three southern African countries; Health Research Policy and Systems 2009, **7**:31 doi:10.1186/1478-4505-7-31 | 2009 | Exploring and comparing the perceptions of stakeholders involved in research and policy making in the two cases (use of magnesium sulphate and insecticide treated nets) in Mozambique, South Africa and Zimbabwe. |
| 29 | Anthony Costello; | Moving to research partnerships in developing countries, 2000, BMJ; 321:827 | 2000 | To study principles behind investment in research in developing countries. |
| 30 | Jansen MWJ, van Oers HAM, Kok G, de Vries NK;, 8:37 | [Public health: disconnections between policy, practice and research](http://www.health-policy-systems.com/content/8/1/37) *Health Research Policy and Systems* 2010 | 2000 | To study conflicting aspects as disconnections in policy, practice and research work cycles |
| 31 | Woelk G, Daniels K, Cliff J, Lewin S. et al | Translating research into policy: lessons learned from eclampsia treatment and malaria control in three southern African countries; 2009, *Health Research Policy and Systems*  **7**:31 | 2009 | To explores and compare the perceptions of stakeholders involved in research and policy making in the two cases (the use of magnesium sulphate (MgSO4) in the treatment of eclampsia and severe pre-eclampsia in pregnancy; and the use of insecticide treated bed nets and indoor residual household spraying for malaria vector control) in Mozambique, South Africa and Zimbabwe. |
| 32 | Kristina Jönsson K.,, Göran Tomson,, Christer Jönsson,, Sengchanh Kounnavong & Rolf Wahlström; | Health systems research in Lao PDR: capacity development for getting research into policy and practice, 2007, *Health Research Policy and Systems*, 5:11 | 2007 | To explore perceptions about how findings from Health Systems Research projects are or should be communicated and used for decision-making together with capacity development of the participants. |
| 33 | Albert MA, Fretheim A, Maïga D; | [Factors influencing the utilization of research findings by health policy-makers in a developing country: the selection of Mali's essential medicines; 2007,](http://www.health-policy-systems.com/content/5/1/2) *Health Research Policy and Systems* 2007, 5:2 | 2007 | To explore factors influencing utilization of research by examining the policy-making process for a pharmaceutical policy, common in developing countries; an essential medicines list. |
| 34 | McMichael C., Waters E., & Volmink J; | Evidence-based public health: what does it offer developing countries?; 2005, Journal of Public Health VoI. 27, No. 2, pp. 215–221 | 2005 | To discuss issues surrounding the relevance of evidence-based public health and systematic reviews to the health of developing countries. |
| 35 | Chigozie J., Uneke, Abel E., Ezeoha, Chinwendu D., Ndukwe, Patrick G; et al | Development of Health Policy and Systems research in Nigeria: Lessons from developing countries’. Evidence-Based Health policy making process and practice; Healthcare Policy, 6(1) 2010: 109 - 126 | 2010 | To identify challenges and the potential intervention strategies to Health policy and systems research evidence use in policy making in Nigeria; by policymakers and other stakeholders in the health sector |
| 36 | Harpham T. & Tuan T, | Lessons from the field: from research evidence to policy: maternal health care in Viet Nam: Bulletin of the World Health Organization; 2006 84 (8); 664 – 668 | 2006; | To analyze how and whether evidence was used to develop health care policies for people with mental illness in Viet Nam |
| 37 | Moseley, A. and Tierney, S. | Evidence-based practice in the real world; 2004, Policy Press - *Evidence and Policy: A Journal of Research, Debate and Practice*, Vol. 1, No. 1, pp.113-120 | 2004 | To present the practical problems of implementing evidence-based practice (EBP) |
| 38 | Hornby, P. and Perera, H.S.R. | A development framework for promoting evidence-based policy action: drawing on experiences in Sri Lanka, 2002, *The International Journal of Health Planning and Management*, Vol. 17, No. 2, pp165-183. | 2002 | To explore the implication and the use of indicators to support evidence based policy decision-making, and; the complexity of doing so in Ministries of Health that are undergoing some form of health sector reform. |
| 39 | Oxman AD. Vandik PO et al | SUPPORT Tools for evidence-informed health Policymaking (STP) 2: Improving how your organization supports the use of research evidence to inform policy making; 2009, *Health Research Policy and Systems, 7(suppl 1):S2* doi:10.1186/1478-4505-7-S1-S2 | 2009 | To address ways of organizing efforts to support evidence-informed health policy making |
| 40 | Lavis JN, Guindon GE, Cameron D., et al | Bridging the gaps between research, policy and practice in low-and middle-income countries: a survey of researchers; 2010 *CMAJ 182 (9): 350 =- 360* | 2010 | To study efforts to bridge the gaps between research, policy and practice in 10 low- and middle-income countries |
| 41 | Lavis JN., Oxman AD, Moyniham R & Paulsen E. | Evidence informed health policy 1 – Synthesis of findings from a multi-method study of organizations that support the use of research evidence; 2008, *Implementation Science; 3:53* doi:10.1186/1748-5908-3-53 | 2008 | To identify organizations around the world, especially in low and middle income countries that are in some way successful or innovative in supporting the use of research evidence in eth development of clinical practice guidelines, health technology assessment and health policy, and describing their experiences. |
| 42 | Lavis JN., Paulsen E., Oxman AD & Moyniham R. | Evidence informed health policy 2 – Survey of organizations that support the use of research evidence; 2008, *Implementation Science; 3:54* doi:10.1186/1748-5908-3-54 | 2008 | To identify organizations around the world, especially in low and middle income countries that are in some way successful or innovative in supporting the use of research evidence in eth development of clinical practice guidelines, health technology assessment and health policy, and describing their experiences |
| 43 | Sevene E, Lewin S., Mariano A., et al | System and market failures: the unavailability of magnesium sulphate for the treatment of eclampsia and pre-eclampsia in Mozambique and Zimbabwe; 2005;BMJ 331: 765 - 769 | 2005 | To describe problems with the registration, approval, acquisition, and distribution of magnesium sulphate, and hence its availability to clinicians, in Mozambique and Zimbabwe |
| 44 | Oxman AD, Lavis JN, Fretheim A.,& Lewin S | SUPPORT Tools for evidence-informed health Policymaking (STP) 16: Using research evidence in balancing the pros and cons of policies; 2009 *Health Research Policy and Systems, 7(suppl 1):S16* doi:10.1186/1478-4505-7-S1-S16 | 2009 | To inform judgments about the balance between the pros and cons of policy and programme options. |
| 45 | Innvaer S, Vist G, Trommald M, Oxman A; | Health policymakers’ perceptions of their use of evidence: a systematic review; 2002; *Journal of Health Services Research Policy; 7(4):239 – 44* | 2002 | To summarize the evidence from interview studies of facilitators of and barriers to, the use of research evidence by health policy-makers |
| 46 | Tomson G, Paphassarang C, Jonsson K, Houamboun K, Akkhavong K, Wahlstrom R; | Decision- makers and the usefulness of research evidence in policy implementation – a case study from Lao PDR2005; Social Science and Medicine 61(6):1291 – 9 | 2005 | To explore decision-makers’ knowledge of the National drug policy and their attitude regarding the usefulness of health systems research for policy implementation. |
| 47 | Varkevisser C.; Mwaloko G., * Le Grand A., | Research in action: the training approach of the joint Health Systems Research Project for the Southern Africa Region.; 2001; *Health policy and planning; 16(3):281-291* | 2001 | To describe the approach of the joint health system research project over the first 10 years of it’s operation, and its major strengths and limitations. |
| 48 | Oxman AD, Lewin S. Lavis JN, & Fretheim A. | SUPPORT Tools for evidence-informed health Policymaking (STP) 15: Engaging the public in evidence-informed policymaking; 2009 *Health Research Policy and Systems, 7(suppl 1):S15* doi:10.1186/1478-4505-7-S1-S15 | 2009 | To address strategies to inform and engage the public in policy development and implementation. |
| 49 | The Council of Health Research and Development working group on Research to Action and Policy. | COHRED issues paper: Lessons in Research to Action and Policy – Case studies from seven countries. 2000; Geneva: | 2000 | Case studies on Research to Action and Policy in seven countries |
