# Supplementary material for: Research, evidence and policymaking: the perspectives of policy actors on improving uptake of evidence in health policy development and implementation in Uganda
Source: BMC Public Health. 2012 Feb 9;12:109. doi: 10.1186/1471-2458-12-109 (PMC3305540; doi:10.1186/1471-2458-12-109)
Supplement: Additional file 2 — Interview guide. [file 1471-2458-12-109-S2.DOCX]

**Appendix 2: Interview guide:**

Through review of literature, several studies have documented favorable factors and barriers to uptake of research/evidence at the different stages of the research process. I would like to get your views on these parameters. My focus is on public health polices and NOT clinical practice.

I have defined research broadly to include, research studies (both published and unpublished) and monitoring and evaluation studies, undertaken within the country by research institutions, universities, donors, civil society and government statistical units and; Ministry of Health reports.

You have been selected as one of the respondents because of your involvement in policy development/ you have undertaken research in this area.

May I use the recorder to record your responses?

Name of the interviewer:

__________________________________________________________

Date and time of the interview:

__________________________________________________________

*Details of the respondent:*

Name of respondent:

__________________________________________________________

Title of respondent/Designation:

__________________________________________

Stakeholder group/subsector respondent belongs to:

____________________________________________

Could you please briefly describe to me your current role and work responsibilities in this organization/institution *(probe for qualifications, research/policy involvement, and work experience*

Duration in that position: ________years; ___________months;______________

***.***

1. This interview is about the subject of knowledge translation (KT) or getting research into policy and practice (GRIPP). What is your general understanding and view on KT/GRIPP?
2. I suppose that attempts/efforts have been undertaken in your institution / country to get research translated into policy and practice. Could you elaborate on this? If so, which activities and processes were implemented? What were the results? Please provide some more information and cite concrete examples.
3. Have you been *personally* involved in any sort of knowledge translation activities? What is your own experience with that? Was it successful or not? And why?
4. In your view and experience, what would generally speaking be favorable (or facilitating) factors for improving uptake of research into policy and practice/implementation? *(probe for the factors at the different stages/areas in line with the MRT)*
5. What would be in your view the most important facilitating factors? Could you possibly rank what in your analysis would be the top three factors in level of importance? – starting with the most important to the least important. Please explain your ranking.
6. What in your view/experience are possible barriers to research uptake?
7. Can you rank these barriers in the level of importance? - starting with the most significant to the least significant. Could you come up with a top three? Please explain your ranking.
8. Who are in your view the main stakeholders in the process of improving research uptake? (Donors, politicians, the media, civil society, the community, academics, policymakers???) And what role(s) do they play in your view? Positive roles? Negative roles?
9. What are according to you the priorities in terms of creating an environment *conducive* for research results to be translated in policy and practice? What would be your general suggestions and/or recommendations?
10. Is there any final comment you would like to make?

Thank you for this interview.

**In case of any further questions or clarifications; please contact:
Juliet Nabyonga**

**E: mail:** [**julienabyonga@yahoo.com**](mailto:julienabyonga@yahoo.com)

**Phone no. 0772 488 596**
